# Difficult clinical management of groove pancreatitis: a case report

**DOI:** 10.31744/einstein_journal/2025RC1216

**Published:** 2025-10-13

**Authors:** Matheus Marcelino Dias, Marcelo Bruno de Rezende, Rielly de Sousa e Silva, Vanderlei Segatelli, Adriano Tachibana

**Affiliations:** 1 Hospital Israelita Albert Einstein São Paulo SP Brazil Hospital Israelita Albert Einstein, São Paulo, SP, Brazil.

**Keywords:** Abdominal pain, Pancreatitis, chronic

## Abstract

Groove pancreatitis is a rare form of chronic pancreatitis that affects the anatomical area between the head of the pancreas, medial wall of the second part of the duodenum, and common bile duct, known as the groove area. We present the case of a 40-year-old female patient with recurrent acute abdominal pain, vomiting, inappetence, and weight loss over several months. Diagnostic imaging revealed obliteration of fat in the pancreaticoduodenal groove and densification of the adjacent adipose planes, along with an infiltrative and stenosing lesion in the duodenum. Despite negative biopsy results, the patient's symptoms persisted, leading to a multidisciplinary decision regarding surgical intervention. Initially, a less-invasive robotic gastroenteric anastomosis was performed; however, this did not yield the desired outcome. Consequently, after further episodes of pancreatic inflammation, a more extensive pancreaticoduodenectomy (Whipple procedure) was performed. The patient showed significant clinical improvement postoperatively, with no recurrence of symptoms. Groove pancreatitis poses a diagnostic challenge owing to its nonspecific clinical presentation and imaging findings. Although management typically involves dietary changes, medication, and endoscopic interventions, surgical intervention may be necessary in cases of recurrent symptoms or complications. This case highlights the importance of a multidisciplinary approach in managing groove pancreatitis and underscores the potential efficacy of surgical intervention in achieving symptomatic relief and improving quality of life, even in atypical patient demographics.

## INTRODUCTION

Groove pancreatitis (GP), a rare form of chronic pancreatitis, occurs in the area between the pancreas and duodenum, known as the groove area. It is classified into two types: pure, when confined to the groove area, and segmental, when extending into the pancreatic head.^(1,2)^ Treatment options include clinical management, endoscopic procedures, and surgical interventions, with multidisciplinary evaluation and shared decision-making playing a crucial role.

## CASE REPORT

A 40-year-old female patient presented with acute abdominal pain in the right flank, radiating to the right iliac fossa, accompanied by episodes of vomiting, loss of appetite, and abdominal distension. Since October 2022, she had made recurrent visits to various emergency rooms.

**Figure 1:**
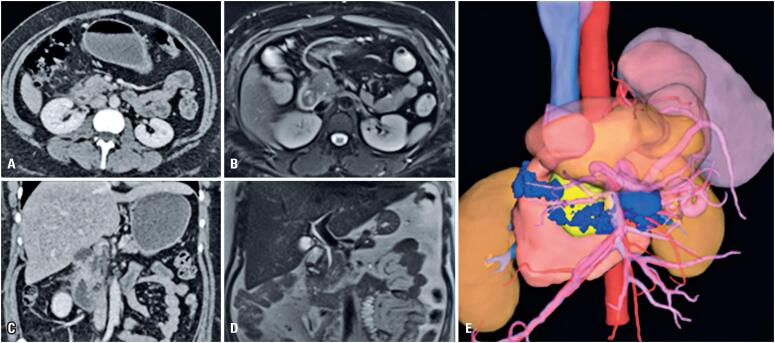
Portal phase of contrast enhanced abdominal CT, depicted in both axial (A) and coronal (C) views using the abdominal window. The images show irregular parietal thickening of the first and second portions of the duodenum, along with increased density of the surrounding fatty tissue. These findings are also evident in the corresponding abdominal MRI: the axial view with fat saturation using the T2 Blade sequence (B) and coronal view using the T2 Haste sequence (D). The 3D reconstruction of the CT (E), visualized in blue, reveals inflammatory tissue affecting the duodenum, leading to stenosis in the second portion and extending into the mesentery, which also causes stomach dilation. Pancreas is displayed in yellow

**Figure 2:**
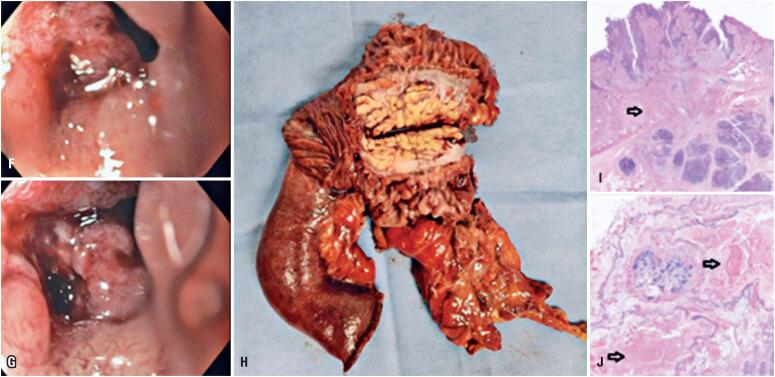
Digestive endoscopy images (F and G) showing an infiltrative and stenosing lesion from the upper flexure to the second portion of the duodenum, where biopsies were performed. The postoperative specimen from the pancreaticoduodenectomy (H) displays the first and second portions of the duodenum and part of the head of the pancreas, with infiltrative fibrous tissue affecting the space between these anatomical structures. The anatomopathological study revealed fibrosis and hyalinization with chronic inflammatory cell infiltrate (I, arrow) and areas of necrosis (J, arrows) present in the muscular layer of the duodenum and involving the adjacent pancreatic parenchyma (HE)

In March 2023, she was admitted to our emergency department with a clinical condition that affected all aspects of her life. She was unable to work or care for her young son and lost weight due to her inability to eat. Initial investigations included abdominal computed tomography (CT), which revealed obliteration of fat in the pancreaticoduodenal groove and densification of adjacent adipose tissue in the first and second portions of the duodenum. These findings were corroborated by abdominal magnetic resonance imaging (Figure 1). Endoscopy demonstrated an infiltrative and stenosing lesion in the duodenum, with biopsy negative for cancer (Figure 2). Other differential diagnoses, such as perforation by foreign body, duodenitis, and pancreatic neoplasia, were ruled out based on clinical history, imaging, and laboratory tests. Considering the patient's young age, we opted for clinical management without surgery to control her symptoms. During her initial hospitalization, she received total parenteral nutrition and analgesia, and progressed well until discharge, with dietary adjustments and somatostatin treatment. After experiencing six uncontrolled recurrence episodes that severely affected her quality of life and work performance, a shared decision was made to pursue the least invasive surgical approach. Robotic Roux-en-Y gastroenteric anastomosis was performed to manage the patient's symptoms. Despite this, she developed new episodes of pancreatic inflammation, and four months later, she underwent pancreaticoduodenectomy (Whipple procedure). There were no postoperative complications or blood transfusions, and the patient had a short stay in the intensive care unit. The cavity inventory (Figure 2) showed that the pancreas was hardened throughout, particularly in the head, with a locoregional inflammatory reaction and adhesion of the epiploon to the fat. The specimen was sent for an anatomopathological analysis (Figure 2), which confirmed the diagnosis of GP. Surgery resulted in significant improvements, with no symptom recurrence, allowing the patient to resume her daily activities and work.

The study was approved by the Research Ethics Committee of *Hospital Israelita Albert Einstein* (CAAE: 86927025.7.0000.0071; #7.563.607).

## DISCUSSION

Groove pancreatitis was initially described in 1973 by Becker, who used the German term "Rinnenpankreatitis."^(3,4)^ Later, in 1982, Stolte et al. referred to the condition as GP, as it affects the anatomical area between the head of the pancreas, medial wall of the second part of the duodenum, and common bile duct, commonly known as the groove area.^(1,2)^ The pathogenesis of GP involves the fibrosis of the paraduodenal tunnel, leading to altered pancreatic secretions and pancreatitis.^(1,2)^

In contrast to the reported case, GP typically affects men in their fifth decade of life and is often associated with a history of alcoholism or smoking. The symptoms include upper abdominal pain, diarrhea, weight loss, postprandial vomiting due to duodenal obstruction, jaundice, and cholestasis.^(1,2,5)^ GP is particularly important in the differential diagnosis of pancreatic ductal adenocarcinoma and duodenal carcinoma.^(5)^

Diagnosing GP is challenging because of the nonspecific nature of laboratory markers.^(6)^ However, tumor markers such as carcinoembryonic antigen and carbohydrate antigen typically remain at normal levels.^(7)^ In difficult cases, fine-needle aspiration via endoscopic ultrasound has proven to be effective in distinguishing GP from malignant tumors, with high sensitivity, specificity, and predictive values (90%, 100%, 100%, 92.86%, and 95.65%, respectively).^(8)^

As demonstrated in the CT findings of the present case, imaging of GP may reveal the thickening of the duodenal wall, enlargement of the pancreatic head, and biliary duct stricture or dilation.^(3)^ These findings are difficult to differentiate from those of other conditions. CT findings often correlate with histological features,^(1)^ with the pure subtype showing scar tissue as a hypodense, laminar mass between the duodenum – near the minor papilla – and the pancreatic head.^(1)^

Magnetic resonance imaging is considered the preferred diagnostic method,^(9)^ with a diagnostic accuracy of 87.2%.^(1)^ It typically shows a mass that is hypointense on T1 and hyperintense on T2 weighted images compared with those of the pancreatic parenchyma.^(1)^ In our patient, MR cholangiography also demonstrated duodenal and pancreatic duct obstruction due to the progression of chronic pancreatitis with fibrosis.

The clinical management of GP includes dietary changes and, if applicable, the cessation of smoking and alcohol consumption. Medicinal options include analgesics and somatostatins.^(1)^ The endoscopic approach offers less invasive treatment options, such as cystenterostomy, pancreatic or biliary sphincterotomy with stent placement, and duodenal dilation. However, complications such as stent migration or occlusion may occur^(1)^, and patients may require multiple procedures.^(10)^ This limitation influenced our patient's therapeutic decision, as she sought to improve her quality of life without frequent hospitalizations due to recurrent symptoms.

A systematic review by Ukegjini et al.^(10)^ analyzed 27 studies covering 920 patients and found that 74.1% of the patients were treated conservatively, with 35.6% achieving complete resolution. Patients with refractory disease eligible for endoscopic treatment had a success rate of 55.2%. Ultimately, 54.7% of the patients were treated surgically, with 69.6% of these patients experiencing complete resolution. Only 8% experienced clinical recurrence after surgery.

Managing these cases is challenging, especially in young women, as the dilemma lies between attempting clinical or endoscopic management, which are less invasive, and opting for surgery, which has been proven to be effective. In cases of GP that are difficult to manage clinically, have an uncertain diagnosis, or require a reduction in recurrent symptoms, the gold standard treatment is pancreaticoduodenectomy.^(10)^

## CONCLUSION

Groove pancreatitis presents significant challenges in both diagnosis and management. This case illustrates an atypical epidemiological instance occurring in a female patient without a history of smoking or alcoholism, who developed duodenal stenosis with recurrent symptoms that necessitated major surgery for definitive treatment.
